# Experiences of longing in daily life and associations to well-being among frail older adults receiving home care: a qualitative study

**DOI:** 10.1080/17482631.2020.1857950

**Published:** 2020-12-17

**Authors:** Jessica Hemberg, Fredrica Nyqvist, Venke Ueland, Marina Näsman

**Affiliations:** a Department of Caring Sciences, Faculty of Education and Welfare Studies, Åbo Akademi University, Vaasa, Finland; b Social Policy Unit, Faculty of Education and Welfare Studies, Åbo Akademi University, Vaasa, Finland; c Faculty of Health Sciences, University of Stavanger, Norway

**Keywords:** Caring, daily life, experiences, frail older adults, home care, longing, well-being

## Abstract

**Purpose**: All over the world, communities face the challenge of maintaining well-being among older adults. More specifically, understanding the resources required to establish and maintain well-being among community-dwelling older adults is an essential issue. Although longing from a caring science perspective is considered a driver for well-being, it has not yet been investigated among frail older adults. The aim of this study was to explore frail older adults’ experiences of longing in daily life and the relation between longing and well-being from a caring science perspective.

**Method**: The study uses a hermeneutical approach and follows a qualitative explorative design. The data comprises texts from 17 interviews with frail older adults and was analysed by content analysis.

**Results**: The results uncovered three themes: *Longing for social contacts; Longing for nature* and *Longing creativity, aesthetics, and music*. Longing was positively related to well-being when the older adults were able to fulfil their longings.

**Conclusion**: This study provides an understanding of the mechanisms of longing among frail older adults. Longing, here, is an inner resource for setting into motion the transition towards well-being. Further studies could focus on how frail older adults can be supported to combat the negative forms of longing in daily life.

## Introduction

Ageing populations all over the world face the challenge of maintaining their well-being (Nieboer & Cramm, 2018a). The ability for older adults to remain independent for as long as possible has become all the more vital, since it has the potential to ease the demands on the social and health-care system in society (Barlow et al., 2002). Older adults can feel safe, independent, and autonomous when they are in a familiar and meaningful environment, which is the main reason why they wish to age-in-place and remain in their homes throughout their lifetime (Dahlin-Ivanoff et al., 2007; Tan et al., 2015). In this sense, understanding community-dwelling older adults’ recourses for maintaining and establishing their well-being becomes an essential issue. The well-being of older adults is thus a growing subject matter of importance in policy and in debates regarding economic issues. As such, the improvement of well-being is seen as a strategic societal objective (Steptoe et al., 2015).

From a caring science perspective, longing can function as an internal resource or trait towards well-being and health (Eriksson, 2018; Ueland et al., 2018). However, when longing as a driving force (Eriksson, 2018) cannot be fulfilled, the older adults feel that their dignity is disrespected, which can create a negative longing instead. By extension, this type of negative longing can eventually extinguish the spark of life. Although there is a lot of research on well-being, not much has been made on longing (Ueland, 2013; Ueland et al., 2020, 2018) and especially not regarding the relation between longing and well-being in frail older adults. Frail older adults are a study group of specific interest considering that physical limitations might prevent or limit underlying resources, such as longing, which are needed for maintaining well-being. This frailty makes older adults more at risk of encountering a wide array of detrimental outcomes (for instance: disability, falls, hospitalization, care-home admission, mortality) due to subtle and progressive physical transformations (Andrew et al., 2012).

At the same time, frail older adults, as a vulnerable group, have more trouble than the general older population in attaining a particular level of well-being when confronted with an array of changes in life (Näsman et al., 2019; Nieboer & Cramm, 2018a). Pennbrant and Karlsson (2019) underline that in order to promote participation, well-being, and dignity, it is crucial that frail older adults are part of a caring culture and relationship in order to meet their care needs and ensure high-quality care. In this study, we therefore apply a caring science perspective to increase the understanding of the significance of longing for well-being among community-dwelling frail older adults receiving home-based care. The next section is a literature review of previous research focusing on well-being among older adults, after which we discuss the concepts of longing and well-being in accordance with caring science theory.

## Previous studies on older adults’ well-being

In this study, we focus on the perceived, subjective, aspect of well-being in frail older adults. This focus aligns with the view of well-being described in the caring science tradition, as a subjective experience that can prevail also in the presence of ill-health (Eriksson, 2018). As can be seen in the text, there are several concepts being used which refer to well-being. Therefore, to avoid confusion, we choose to use an inclusive approach, meaning that the concepts used are the same as those the authors themselves use in their research. Still, we are aware that many of the concepts below are mutually exclusive.

Subjective well-being (SWB) is a wide-ranging concept that encompasses both a cognitive element (cognitive well-being) and an affective element (affective well-being), which in turn is often also divided into positive (happiness, engagement, and joy) and negative (worry, fear, uneasiness) affect (Diener, 1984; Lucas et al., 1996). SWB may, thus, involve the assessments individuals make of their own lives, including cognitive judgements, e.g., satisfaction with life, as well as assessments based on emotions. SWB also implies a state of mind (Smith et al., 2002; Tov & Diener, 2009).

Previous research shows that SWB in old age can be affected by many aspects of life, including health-related and social factors. Some of these issues are presented below.

SWB is associated with elements such as the degree of social integration, support networks, and professional activities (Dolan & White, 2007; Herrera et al., 2014; Silva, 2014) as well as social contacts, including family and friends (Kamp Dush & Amato, 2005; Lara et al., 2019). In addition, older adults with partners (Kamp Dush & Amato, 2005), a higher level of education (San Román et al., 2017), and high incomes have a higher status and stimulation levels as well as a higher well-being than those without such resources (Barnes et al., 2006; Lyons, 2009).

In enhancing well-being among frail older adults, it is important to follow-up their financial security, assess and enable their personal relationships and strive to meet their basic needs (Hoeyberghs et al., 2019). According to an interview study conducted by Learmonth et al. (2012), having support and supporting others within the community was important and could enhance frail older women’s sense of belonging and emotional well-being; and *social interaction* was the key factor that enhanced their well-being. *Social connectedness* for older adults can generate feelings of belonging and solidarity (Gallagher, 2012). According to Nieboer and Cramm (2018b), the prevention of a deterioration in well-being may be made by interventions designed to maintain the significant activities of older adults. Engaging in fruitful activities could positively affect their personal development which, in turn, can generate positive outcomes on well-being (Lara et al., 2019).

Research indicates that health is critical for well-being in very old age (Smith et al., 2002). Staying healthy and maintaining autonomy has been highlighted by very old adults themselves as factors enhancing their mental well-being (Lara et al., 2019). However, well-being is limited by chronic illness and functional impairments such as hearing, vision, mobility, and strength, particularly in very old age (Smith et al., 2002). Health affects well-being so that over time, physical illnesses (especially those with severe symptoms) are likely to affect functional health together with the routines of daily life, thus making it more troublesome to take part in social activities in various social contexts. A decreasing functional health has a negative effect on the sense of well-being amongst older adults (Smith et al., 2002). At the same time, research also shows that the gap between objective health, including measures of, for example, activities of daily living, and subjective (self-rated) health amongst older adults increases (Wettstein et al., 2016), suggesting that the path between objective health and well-being is not straightforward.

Besides social and health-related factors, well-being in old age has also been associated with inner resources, such as having a positive outlook on life (Lara et al., 2019) and self-transcendence (Haugan et al., 2013). Considering research on longing in other contexts (e.g., Ueland et al., 2018), we also expected that longing could be such a resource. In this study, longing is thus seen as a driver or strengthener for well-being amongst frail older adults and it was the subjective experiences of these individuals that were in focus. The study is based on Eriksson’s descriptions of well-being and the relationship between longing and well-being (Eriksson, 2018). From a caring science perspective, a sense of dignity as well as well-being could be strengthened when a frail older person’s longing for different aspects of everyday life has been fulfilled. However, to our knowledge, no qualitative studies have previously been conducted regarding longing and its association with well-being in frail older adults, which is why this study brings valuable contributions to the existing well-being literature.

## Aim

The aim of this study was to explore frail older adults’ experiences of longing in daily life and the relation between longing and well-being from a caring science perspective.

## Theoretical framework

This study is grounded in a humanistic tradition, within Eriksson’s theory of Caring Science (Lindström et al., 2011). Well-being is a condition whereby a person can experience their own health positively, irrespective of whether they suffer from illness or disability, thus making it an important human experience (Eriksson, 2018). Well-being is an experience that cannot be seen or determined objectively; only subjectively (Eriksson, 2018). In addition, well-being can be experienced even when a person is seen objectively as having ill-health (Eriksson, 2018).

Longing as a subjective experience might be closely related to health and well-being, since longing may be an expression of a person’s unique inner world (Ueland et al., 2018). Since longing from a caring science perspective can be seen as a driving force for well-being, longing becomes an important aspect in enhancing the well-being of frail older adults.

Eriksson claims that longing is rooted in the primary substance of Caritas, in a person’s inner Ethos (Lindström et al., 2011). She also claims that a person’s deepest longing is “to be love”, that is, to give and receive love (Eriksson, 1987). Love, as a source of power in the movement between suffering and health, can be experienced through encounters with: another person, the self, the abstract other, or with nature (Eriksson, 2018). Love for one’s neighbour is a fundamental substance in the movement of becoming health, and out of the trinity, faith, hope, and love, it is love that has the deepest dignity in terms of providing power, whereby a person can dedicate to themselves the power to become health (Hemberg, 2015).

In this light, longing appears to be a dynamic power of becoming in the movement between suffering and health, striving towards living one’s own original drama (Eriksson, 1994). However, a person’s involuntary unfulfilled longing can lead to experiences of suffering, whilst a longing that can be fulfilled (if the person wishes for it to be fulfilled) can lead to well-being and health. Thus, according to Eriksson (2018), a person’s subjective evaluation of perceived well-being is a sufficient condition for health. Living one’s own original drama, such as living one’s life, might be related to the direction of movement towards life fulfilment, which is determined by longing, i.e., a thirst for something more (Eriksson, 1994). Although a person’s longing can be directed towards a lust or a need, on a deeper level, longing goes towards the realization of one’s potential, reconciliation of life, a deeper understanding of life and finally transcending life (Eriksson, 2007).

Experiencing harmony and that life is worth living might be promoted by a transcendent longing that moves the person towards the ultimate fulfilment, a deeper relation to another, i.e., God or an external power (Eriksson, 2003). A more definite longing might be a journey towards the most evident, the core of life, which means to come closer to an inner entity and holiness as a deeper wholeness of body, soul, and spirit (Eriksson, 2018). The power of longing appears to be released when people remain in a state of tension between a future possibility and uncertainty, irrespective of whether or not the longing will be fulfilled (Ueland et al., 2018). As such, the moment of longing might be a process towards reconciliation, which means that a person might become whole through the process of developing towards health (Ueland et al., 2018).

## Materials and methods

In this study an explorative research design was used. This study was part of a larger project, with the overall aim to explore well-being, social participation, and loneliness among frail older adults receiving home-based care. Several researchers took part in this phase and conducted the interviews. However, the most experienced researcher regarding interview studies conducted the major part of these. A total of 17 interviews were conducted. The interviews were performed by four researchers and were subsequently transcribed by three. J.H. conducted ten interviews, M.N. conducted five interviews, F.N. conducted two interviews and L.B. conducted one interview. The researcher who conducted only one interview did not participate in either the analysis or writing of the manuscript. Afterwards, J.H. transcribed ten interviews, M.N. transcribed five interviews, and L.B. transcribed two interviews. An interview guide was referred to during the interviews, whereby focus was placed on quality of life, well-being, social- and cultural activities, and experiences of loneliness. Three different themes were included in the interview guide: (a) activities and interests (previous and current), (b) social contacts (previous and current) and (c) experiences of loneliness and wishes for and requests from the society regarding how they could provide support for alleviating loneliness amongst frail older adults. For example, these questions were included in the interview guide: ”What provides you with joy of life and well-being in daily life?”, ”What do you lack, dream of or long for in your daily life?”, ”Is there someone who can help you with fulfiling the things you long for?”. The interview guide consisted of semi-structured questions used during the interviews to ensure that all themes were covered. Follow-up questions were left to the interviewer’s discretion, which were dependent on the responses from the participant being interviewed. The researchers made sure that the different themes and questions were raised in an open and neutral way, so as not to influence the responses from the participants.

## Context and participants

The interview data used in this qualitative study was compiled through face-to-face interviews. The themes of these interviews focused on the participants’ quality of life as well as their experiences of daily life. The interviewers came together to discuss the interview methodology before conducting the interviews in order to ensure that there was no variation between the interviewers. A total of 17 participants (12 female and five male), aged 72–95 years and in different life situations, were included in the study, whereby data saturation was considered achieved. All the participants were residing in the same municipality in Finland, and all were receiving municipal community-based home care. The criteria for inclusion in the study was that participants had to be aged 65 years or older, were able and willing to give their informed consent and that they had a desire to share their experiences. The participants were selected in collaboration with the home care staff in the municipality where the study was conducted. Further information on the participants is given in Table I.

**Table I.** Study participants

Participant number	Age	Gender	Marital status	Living alone or with a partner	Form of housing and living place
1	82	Male	Widower	Lives alone	Form of assisted living in a rural area
2	92	Female	Widow	Lives alone	Form of assisted living in a rural area
3	87	Male	Married	Lives with spouse	Own detached home a rural area
4	95	Female	Widow	Lives alone	Own detached home a rural area
5	74	Female	Widower	Lives alone	Own detached home a rural area
6	93	Female	Widow	Lives alone	Own row-house flat in a rural area
7	90	Female	Widow	Lives alone	Own detached home a rural area
8	88	Female	Widow	Lives alone	Own detached home a rural area
9	82	Male	Single	Lives alone	Own detached home a rural area
10	79	Female	Married	Lives with spouse	Form of assisted living facility in the suburbs
11	88	Male	Married	Lives with spouse	Form of assisted living facility in the suburbs
12	72	Female	Married	Lives with spouse	Own detached home a rural area
13	92	Male	Widower	Lives alone	Own detached home a rural area
14	86	Male	Married	Lives with spouse	Own detached home a rural area
15	84	Male	Widower	Lives alone	Own flat in the suburbs close to the city
16	92	Female	Married	Lives with spouse	Own row-house flat in a rural area
17	87	Female	Widow	Lives alone	Own detached house in a rural area

Initially, the home care staff provided individuals who were deemed eligible for the study with oral and written information about the study aims. If they decided to participate, the individuals were then asked to provide their telephone number and sign a form providing their written consent. This was then collected for the researchers by the home care staff. The researchers later contacted the participants by telephone in order to provide brief information about the questions in the interview. In total, the interviews lasted 30–90 minutes. Each interview was digitally recorded and transcribed. The interviews were performed in the homes of the older adults. By providing their informed consent before the interviews, each participant gave their approval for their study participation, data storage, and handling for research purposes.

## Analyses

A latent content analysis, inspired by Graneheim and Lundman (2004), was used to analyse the following text. In total, three researchers took part in the data analysis. One of the researchers (J.H.) conducted the initial analysis individually. The final analysis was made with the other three researchers (F.N. and M.N. and V.U.). First, the data material was approached, read, and analysed openly (using an inductive approach) and close reading to uncover any hidden meaning (the latent part of the analysis). This meant that the text was interpreted using a reading “between the lines” approach. The text was reflected against the caring science tradition used in this study (an overall deductive approach). The researchers’ pre-understanding was that longing was a driving force which had an impact on the well-being of frail older adults, and that not being able to fulfil that longing might cause them suffering. This pre-understanding was articulated and repeated throughout the interpretation in order to ensure it was mastered properly and would not steer the results. In this way, it was possible to challenge the pre-understanding. During the interpretation, the understanding of the parts of the texts was reflected against the whole. In this light, the interpretation has, in the spirit of hermeneutics, been a movement between the whole-parts-whole and between interpretation and understanding in order to discover the substance. In this way, the researchers individually read through the texts several times (the whole) before coming together to analyse and place information into categories (the parts). Afterwards, the interpretation and understanding were compared. The researchers were then able to discuss the categories and verify the findings (reflect against the whole). This entire procedure and hermeneutical movement of interpreting and understanding was made several times. In the results section, the results are presented in main themes rather than only implying what was a positive or a negative longing (please see “Results”). Within all main themes there are examples when longing has positive and/or negative implications for well-being. However, as mentioned above we do not present results according to positive or negative longings, rather according to meaningful themes.

## Ethical considerations

This study is ethically defensible since it might help frail older adults understand their own longing and its association to well-being by expressing their longing in words, and thus support in enhancing the understanding of their well-being and health. The municipality where the participants lived gave permission to conduct the study. All the researchers were aware that it was important to treat the participants with respect and dignity during the study. Difficult emotions that occurred every now and then during the interviews were handled respectfully and the researcher then dwelled with the interviewee for a while just to talk and make sure they felt alright at the end of the interview. This study followed the guidelines defined by the Finnish Advisory Board on Research Ethics (2012).

## Results

The results revealed the following three themes: *Longing for social contacts; Longing for nature*; and *Longing for creativity, aesthetics, and music*. All the themes are described and discussed below. Some types of longing can lead to well-being, while other types can lead to suffering. The results showed that the longing that the frail older adults wished for and could be fulfilled led to well-being. Additionally, the longing that the frail older adults could not fulfil anymore but still dreamed about and recalled in memory (e.g., things they did earlier in life) could lead to well-being if the older adults no longer felt that this longing needed to be fulfilled anymore. However, a longing that was involuntarily left unfulfilled led to ill-being and ultimately suffering, since a person’s curtailed freedom to create their own existence and fulfil a longing was seen as a violation against their unique identity as a person.

## Longing for social contacts

The first theme consisted of the longing older adults had for social contacts. These social contacts consisted of both a longing for social contacts within the home and a longing for social contacts outside the home.

The frail older adults mentioned a longing for a support person (home care personnel or a volunteer) as a social contact. Many of the older adults mentioned that a support person coming to their home would be valuable and would bring well-being to their daily life. *This is how one states this: “yes … I am omnivorous, I also don’t have anything else I’d like to do (here at home), just having a friend to talk to (at home) would be good. Someone who would come home and play cards with me … to have a friend to talk to … who … would cheer me up. It would be important …* (P13) However, some did not want a support person in the home. Still, several of the participants were so bad on their feet or suffered from dizziness or other forms of ill-health that made them unwilling to go away from the home, etc. Therefore, most of the older adults were positive about having a support person visiting their home for social interaction and conversation. Many of the older adults also longed for more spontaneous visits to their homes: someone who would come home and talk for a while, or teach them something new or play games. Another longing among the frail older adults was a longing for contact with someone outside the home who they felt could enhance their well-being. The longing to talk to someone led the frail older adults into action. One participant mentioned the following: *“yes … I usually call someone [to get some joy in life] … that’s probably the best thing. You get to think about something else …”* (P13). The older adults mentioned that a support person who would come to various concerts or to the swimming pool could be good, because the older adults were worried that they would fall because of their unsteady legs. Therefore, the frailty of the older adults limited their ability to fulfil their longings. This is how one explains this:

“ … and then I would also have to go to the swimming pool (for health rehabilitation) … but that also means going alone … so I don’t go … I have probably thought about going in my mind, but I haven’t yet done anything about it (asked a friend to come with) … but maybe if my walking was better, then I would succeed … I just think how I should get to that swimming pool … *”*  (P6)

The longing for a support person to come along could also produce negative effects on well-being if the older adults were not able to fulfil this longing and did not get a support person that could help them to come out.

One participant mentioned that the longing for friends was great, which had a positive effect on their well-being in everyday life. When they thought about friends and if they had the opportunity to cure that longing by meeting their friends every now and then: *“Yes, it’s the people I miss the most, yes … (laughter) I probably have a lot of friends …”* (P11) Many of the older adults expressed a longing for social contacts, and for some, this longing could not be fulfilled, whereupon it aroused negative thoughts and suffering. Some participants mentioned that they had the opportunity to visit neighbours if they wanted some social interaction. Others were prevented from going because their health was failing physically or because they felt that their neighbours were too busy with work and/or their own families. One participant mentioned that times have changed so that one can no longer go to each other on spontaneous visits, as it used to be in the past, and that this was something that was longed for and created suffering when that longing could not be fulfilled. Another emphasized that as you get older it becomes harder to keep in touch with your friends and you become isolated.

There was also a longing for deeper friendships, not as a partner, but still as a daily contact, i.e., someone with whom you could sit and talk. This was felt negatively when the longing could not be fulfilled. If the friend or relatives are gone, then that longing cannot be fulfilled, as one participant expressed:

“Well, I usually think I could have a friend … but not … [pause] I don’t want someone new, who would be here all the time … No, but a friend who could talk … And that … the priest [name] … he turned 95, but now he’s gone … he lived close by … so he came in now and then and he liked my company he … I talked and he liked old things and stuff, so he thought it was funny that I was interested in things like that. But now he’s gone … And I made him coffee … here he sat … *”*  (P1)

The participants also expressed a longing for meeting like-minded people, preferably at the same age as themselves. One male participant mentioned that the lack of social contacts is great, but that is because his closest friends have past away and he found it difficult to establish new friendships in old age. One participant mentioned the following*: “Well, in a way you are missing everyone (former friends), but it is difficult to get in touch with them, when they are gone, they are gone …”* (P3). This older male participant also mentioned that it is difficult to find new friends and that those who come from the home care service are so young, which makes it difficult to form friendships. Great exchange and well-being was gained by spending time with the same age group.

The participants also mentioned that older people often do not have the energy to get involved, and they felt that the “threshold” for them to contact new people or knock on a door and call a neighbour was too big in old age. One participant also mentioned that it is difficult to succeed in making new friends as one gets older, and that failure to create and retain friends as a frail older adult can adversely affect well-being. Another participant turned out to be reconciled with the circumstances of life and found that it is part of life that one does not have as much social contact as one gets older.

Although the home care personnel was seen as an important element in everyday life for the older people, they nevertheless mentioned that a great exchange was obtained from social contacts who were also more confident, from family and relatives, for example, and that was what provided the most emotional support and trust. Several participants expressed a longing to meet others, and a sense of positivity in their lives whenever it happened. Grandchildren also brought great joy and a source of something to long for in the everyday lives of some of the older people. Their well-being could be enhanced if they were able to fulfil this yearning by seeing their grandchildren on a regular basis. *“When the grandchildren come … it’s probably the highlight of the day when they come … it’s so nice …”* (P14). But when that longing could not be fulfilled, the longing created suffering: *“These (grandchildren) who live abroad I miss … well, yes, I have mourned a lot … and this daughter is the only girl I have … [sobs]”* (P6)

However, some participants also mentioned that they experienced social isolation in the countryside, and they did not have the same opportunities for social interaction. They also mentioned that they could not be themselves or feel alive because of this and thus had a longing to get together and talk to people. Being unable to realize that longing was described as having a negative effect on their well-being. Here’s how one participant who lived with her husband expressed this:

“Firstly, I wouldn’t want to live here. I would like to live where there’s a little more happening … I am so, trapped here … and soon I have no goals left. Yes, you become more and more indifferent … [silences … whispers] I don’t know … I liked to talk to people, have company with people, have fun … to hang out … read a book or a newspaper and discuss things … I would like to talk to people! To discuss things and be alive!” (P2)

As mentioned earlier, some of the participants stated that they did not have a need for social contacts, but displayed also an underlying desire to meet people. One participant said: *“Well, I don’t know … (don’t have contacts you had before in life, or new) … no, but it may happen that it would be good (to have social contacts) … Well I’m probably that (interested in people) …”* (P4). Another participant who seemed to be able to live alone also began to express that he still had an inner desire to have a close acquaintance or friend, which was also expressed as a suffering, because he lacked the strength and ability to fulfil that desire: *“You would have to get someone who you could, for example, live with … and have as a close friend really … But it doesn’t work like that anymore … You are too far away …”* (P3).

Several of the older adults mentioned that they would like to go somewhere as a group, because it would stimulate well-being and enable them to meet new people or form new friendships. One participant, on the other hand, mentioned that she did not want to have much social interaction with other people. Another participant expressed some ambivalent feelings and said that he had no desire for new contacts anymore, but expressed himself like this: *“Yes, well, I don’t know (don’t think it’s fun to get to know new people) … Actually not (don’t need it) But I have nothing against it either directly”* (P5)

It appears that when an older adult lacks the strength and ability to fulfil the longing for socialization, it is detrimental to well-being, and can lead to despondency and boredom, which gives testimony to the following:

“Yes, but unnecessarily long (in life). That is what you think about most, when you have come so unnecessarily far, completely unnecessarily … When you can do nothing more … Well, you can’t do anything more either. It’s the energy that has gone at the same time so … No, when you are exhausted, then you are exhausted. Yes, you’ll be without questions and everything … and the interest disappears for everything …” (P3)

## Longing for nature

The second theme consisted of longing for nature. The participants expressed that they longed to spend time outdoors in the nature, viewing this as a source of strength in daily life. Things mentioned in this respect included getting fresh air and enjoying evening strolls as a way to unload and recharge the batteries for the next day. Well-being was had by staying outdoors and getting stimulation through new situations. One participant said the following:

“ … if they have any extra time then they (the home care personnel) will come and go with me … so I think it’s good that I get some exercise! So the home care nurses come out with me … not every day, but I’m happy when they come, I get fresh air and move my legs … and you get some new impressions when you go out … ” (P12)

For many, the longing for summer was a positive driving force. One participant said the following: *“Yes, if it was beautiful weather, I would like to go out … and be out … because I have always been happy out there in the fresh air …”* (P1). Those who could go out used to do it regularly every day, as one of the participants said: *“Well I can’t do that much nowadays … I usually go out and walk and … and read newspapers and go for food and coffee and stuff …”* (P9). Some participants enjoyed flowers and wildlife in the nature, and the longing for it had positive effects on their well-being. The nature allowed those who could be attracted to get themselves outdoors in everyday life. Here’s how one participant expressed this:

“And so my birds, who fly here … they like me … and in the summer I like wild flowers … and when we have been on a few trips, they usually look so strange to me, when I walk by myself …. by the side of the ditches and I’m interested in flowers” (P1)

Some participants expressed a longing for the summer, as in previous years they once had the opportunity to enjoy sitting outdoors in their yards in the summertime and be close to the nature. But now physical ailments prevented them from doing this, which was expressed as something hindering their well-being. This longing was felt as something negative because their longing could not be fulfilled. The participants mentioned that having to wait for a support person to come and help them get outdoors was a painful experience, since it was deemed important to be able to go out whenever the feeling arose: *“Well, when you want to go out yourself … [it is important to be able to go whenever you want] …”* (P16). The elderly also testified that the fulfilment of the longing to go out to sea and visit the archipelago would have a positive impact on their well-being, as this is what they did earlier in life. However, some cited physical health limitations as an impediment towards their ability to fulfil their longing to be outdoors, which was detrimental to their well-being:

“And I’m still waiting for summer to come and being able to sit out there … but I can’t go that far, my leg gets stiff and sick. And that has hampered me a lot, I was able to ride a bike before. I adapt to my age and body when it comes to what it can do … I have to do what I can and also accept what I can’t do like before. In the past, I cycled, but I can’t bike because of my knees, I have arthritis in my knees, so it’s not like walking around. And that was sad, because I used to ride the bike a lot.” (P16)

Being able to go out alone was experienced by the frail older adults as so meaningful, that it was appreciated even more than being allowed to have company, as one participant explained: *“I can manage [without company] if only I could get out and go …”* (P3).

## Longing for creativity, aesthetics and music

The third theme consisted of longing for music, aesthetics, and creativity. Well-being in daily life for older adults was enhanced by being able to fulfil a longing for listening to music, pottering around with arts and crafts, and other kinds of creativity. Well-being was also experienced when it was possible to fulfil a longing to read a book, or do something creative. Creativity for a frail older adult could, for example, be to decorate or refurnish one’s own home. Well-being in daily life, according to the older adults, also came from having a home in which one thrived and had one’s personal possessions. One participant said the following:

“The most important thing is probably that I have my health … and that I am clear in my head, so to speak … and then it is very important that I can have a home that I enjoy. But I can imagine a much smaller home than what I have today. But it should be pleasant enough. … I’ll probably get some work done and decorate it. … it should look nice … For me, that’s important.” (P6)

A longing to make things aesthetically pleasing could be a source of well-being in daily life, according to the participants. In addition, simply looking at beautiful things could be another source of well-being, such as watching gardening programmes on TV. Thus, *beauty* was mentioned by the older adults as a source for well-being. Here art was mentioned as being an important aspect for bringing well-being to their daily life.

Having the opportunity to fulfil an inner longing to create something beautiful was also associated with well-being. This is how one participant stated this: *“Yes, I have tried to be good there, with needlework … it has been my interest … and so I have done … I would still be like that … I am not an artist, but I have enjoyed painting …”* (P1)

According to the participants, well-being in daily life was also enhanced through music. The participants mentioned that they often longed to listen to music, since this could make them relax and enjoy the moment. They felt that listening to music was a way to renew their strength. The older adults also said that their bodies liked the rhythms in the music. Also, the music may provide a sense of community and security. This is what one participant said:

“ … It was a long time and then I had the music. Oh, it was nice … not at that time, because soon I started to be up and … but that is probably my rehabilitation it is probably the music … It just feels so wonderful inside … I enjoy … relax … and of course … there are some rhythms, then my body follows along … so that’s fun … ” (P6)

Another participant mentioned that she earlier enjoyed dancing together with her husband and that it now provided her with well-being when she remembered those times. She said:

“You know what, we went to the dance! … We danced, the old man and I, and we went together … there were no special couples so, we went together and danced and had fun. … But that was it … then. That was how it was then.” (P10)

Succeeding in fulfiling their longing to solve crossword puzzles, read newspapers, or books was another way in which well-being could be strengthened. Some of the older adults did not have such a large social network or did not feel a need to be with people. One said: *“But I am not really a sociable person so I might as well be alone. I like to read. … It almost gives me more [enjoyment] if I have a good book or something interesting to read.”* (P16)

An important part of well-being was considered to be able to experience that you could do things yourself, as one says: *“On those days when you get nothing done, you start to feel worse because of. it …”* (P11). Participants emphasized that having good eyesight, as well as fine motor skills in their hands, were factors that enabled them to fulfill their longing for an outlet for creativity.

Being able to do what you longed for was considered to be important for well-being. Those who were without their eyesight could not fulfil their longing to do things, e.g., handicrafts, which was something negative. The longing to hand sew was a suffering, as expressed by one participant: *“when you also have something wrong with your eyes … which you cannot … and then you lose the passion when you can’t see …”* (P8). One participant expressed a longing for cognitive stimulants and mentioned that the municipality and home care should remember that it is also important to stimulate the mental and cognitive abilities of older adults, and not just to care for their basic needs. Therefore, it may be important to think about stimulating older adults with things that they themselves are interested in, she said the following: *“Well, that’s the thing to make sure … or to think we’re human. We don’t have a head just to put a cap on, we have something underneath that needs to be engaged … so, I think”* (P2).

## Discussion

The aim of this study was to explore experiences of longing among frail older adults from a caring science perspective and to discover how longing is associated with well-being in their daily lives. The study revealed three themes that were: *Longing for social contacts; Longing for nature*; and *Longing for creativity, aesthetics, and music.*

As mentioned earlier, Ueland et al. (2018) highlight that longing and well-being are related, since longing might be an expression of a person’s unique inner world. In this light, longing can be understood as a driving force for well-being. However, when this longing cannot involuntarily be fulfilled, the person feels violated in their dignity and thus suffers, which can ultimately extinguish the spark of life (Eriksson, 2018). In this study it emerged, in line with Eriksson’s theory, that a longing that can be fulfilled was perceived as something positive, while a longing that was desired but could not be fulfilled was negative and degrading. This means that longing can also be an obstacle (hinderance) if the frail older adults cannot adapt to the circumstances or the situation. The older adults mentioned that frailty in several contexts limited or hindered their ability to fulfil their longing, e.g., to get involved in arts and crafts. The loss of health and functional limitations are clearly risk factors for a decline in well-being (Hansen & Slagsvold, 2012; Smith et al., 2002). Young et al. (2009) suggest that if adaptive strategies are used then successful ageing can exist simultaneously, although older adults have low physical health. Accordingly, promoting the ability of frail older adults to adapt might enable them to fulfil their longings and thereby enhance their well-being. In a study on older women’s experience of health, Tuohy and Cooney (2019) found that, according to the women, the following was important in the process of adaptation: *Balancing needs and support*: in that they appreciated when they were able to be independent and determine how they wanted to live their lives; *navigating in a changing world*: for some older adults it was a difficult phase of life and some women felt sad about what they were losing or could no longer do, also their age limited their opportunities to take care of their health; *Being connected and Involved*: here maintaining relationships was important, and having effort to engage and stay engaged was important, meaning not to stay at home all the time, but to engage in new activities, wanting to remain positive and having responsibilities towards the family (which included remaining engaged). Another important theme was *trying to stay well*: which included being able to do things for one’s self, being active, having a positive body image, and having a healthy weight. In accordance with the caring science theory, the frail older adults would also benefit from being reconciled with life’s circumstances. Eriksson (2003) also mention that a hidden longing can be promoted when a person experiences that life is worth living and this might set a motion into action—towards the fulfiling of this longing. Ueland et al. (2018) also state that longing is entering a movement towards reconciliation of life. In this study, there are things that support parts of Wiklund (2000) and her statements regarding reconciliation as being crucial for achieving health and well-being. Wiklund (2000) states that in all kinds of suffering there are three *acts* that a person must go through, which are, 1) *recognition of the suffering*, 2) *suffering*, (being offered space to suffer and to be able to suffer, meaning daring to encounter one’s suffering) and eventually, assuming that the person has gone through the first two stages, reaching the third and final goal, which is *reconciliation*, where a new meaningful unity is created, which also comprises the “evil”, but since the person is reconciled with life’s circumstances, this so-called evil doesn’t cause suffering, since the person sees meaning in the new unity created, which can lead to health. Here the caregiver plays an important part since they, according to Wiklund (2000), should serve as a co-actor in the drama of suffering and help the suffering person to see and create the new meaning in order to alleviate their suffering and strengthen their dignity.

This study revealed that great benefit and well-being could be achieved by spending time with a similar age group. The longing for social contacts was thus a particularly powerful source of longing for the frail older adults, that could lead to either something positive or negative for their well-being, depending on the possibilities of fulfiling it. Positive and negative longings cannot be fully separated, and in this study it was seen that a longing can be both negative and positive at the same time. It was seen in this study that social longing is a profound and strong request from the frail older adults and that this longing is so strongly rooted in the person. It was found that it is more difficult to rebuild close relationships among frail older adults compared to re-building other longings (the themes of longing found in this study). And the social longing and request for it strongly affected the participants, especially when this longing cannot be fulfilled. This is in line with earlier research, for example, by Dolan and White (2007), Silva (2014), and Herrera et al. (2014), who also highlight the strong relationship between social contacts and well-being. Learmonth et al. (2012), in line with this, stress that social interaction could enhance the well-being of frail older women. Gallagher (2012) similarly highlights that social connectedness for older adults can generate a feeling of belonging and solidarity.

In this study, it was seen as vital for the frail older adults to be able to get out in nature, since they could be stimulated to get new impressions and inputs, they were able to see wild animals such as birds, but also to be able to enjoy the nature, such as flowers and the sea. For some of the participants in this study, having a support person that would help them to get outdoors and enjoy the nature was even more important than having social contacts. Some also mentioned the importance of deciding for themselves when to go outdoors and being able to do so independently. Therefore, the longing for summer was strongly mentioned by the participants, since then it was easier for the frail older adults to go outdoors themselves, rather than in the winter. Another reason for this was also that many of the participants mentioned the longing for flowers and birds as important aspects that would enhance their sense of well-being. With regard to the findings in this study, the fact that the frail older adults had a longing for nature is in line with Burton et al. (2015), who state that it is good for the well-being of older adults that their housing includes a private patio space as well as a large amount of greenery. Older adults also benefit from green spaces, not only by spending time outside but also by seeing it from the inside (Burton et al., 2015). Burton et al. (2015) also state that a green street environment has a probable positive impact on their well-being, if they themselves have none or very little garden space themselves, and if they can view the green street from their home.

The study also revealed that frail older adults have a longing for music, arts, aesthetics and having an outlet for their creativity, which can bring them well-being. The older adults found that it was important to be able to create something beautiful themselves and this was related to their fine motor skills and health-status. Research shows that positive ageing and self-esteem can be strengthened for community dwelling-older adults who listen to and/or play music (Hays, 2005). Older adults reported higher levels of social contact and a sense of belonging and community after having participated in an eight-week singing program (Davidson et al., 2014). Likewise, Liddle et al. (2013) found that arts and crafts contributed to meaningfulness among older women.

We want to highlight that it is important to listen to the older adults’ voices regarding their wishes in daily life and in this way, make them participate because they are experts of their own lives. Our study also revealed that older people themselves could, in most cases, clearly state what they longed for, which were things that were often not too demanding to fulfil, such as outdoor walking or shorter social trips, suggesting the importance of engaging older adults in any discussion. In this study, the frail older adults also longed for more pursuits and meetings that the municipality could arrange. The older adults wanted to participate in arranged programs for them, for example, to have themes about people’s lives, someone would come and tell about themselves or about any travelogue. Some mentioned that they could go to events by themselves or they had a friend who could take them in their car, but far from everyone could get themselves somewhere or had someone who could take them. Accordingly, as previously mentioned, frailty is challenging the older adults in fulfiling their longings. For example, this study revealed that sometimes the longing for social contacts can be exceptionally difficult to re-establish among frail older adults and that it is seldom an easy task to make new connections with people from the same age-group. This is also often the case if the frail older adults’ closest connections have deceased or a particular person is missing from their life. As such, it might be impossible to replace these connections or too difficult for the older adult to make new connections, due to their frailty. Rook (2009) reminds us that with ageing, disruptions may occur in resources and opportunities for support due to illness, the death of a next of kin or other life events. This might create cracks that prevent the older adults from relying on someone for emotional and practical support (Rook, 2009). Several researchers refer to this kind of lack of support as *social frailty* (Bunt et al., 2017; Duppen et al., 2017; De Witte et al., 2013). The risk for social frailty is higher than other dimensions of frailty (e.g., emotional frailty) among community-dwelling older adults and affects older adults on low incomes, are unmarried and have moved during the last ten years (Dury et al., 2017). Since frail older adults might find it more troublesome to take part in activities outside the home, the risk for them experiencing loneliness and social isolation is thus large. In the study by Hemberg et al. (2018), they also found that frailty of health or vulnerability of older adults can be a source that gradually leads to involuntarily loneliness and eventually existential suffering, such as experiences of being homeless in life. Tuohy and Cooney (2019) also report that older women experienced barriers preventing them from participating in different activities in the community due to a low income, ill health and age-related difficulties. Having physically accessible environments and transport can facilitate this participation as well as by having adequate support personnel and equipment (Older & Bolder, 2009). Nieboer and Cramm (2018b) also found that the environment can be a hindrance for well-being, since their study showed poorer levels of well-being among older adults living in less age-friendly communities.

We argue that in home care it is thus vital for health professionals to maintain the older adult’s dignity, which involves taking the frail older people’s perspectives into account, i.e., personality, identity, and self-determination. Söderlund (2004) also mentions that the experience of dignity is thus vital for well-being. Being allowed to be one’s own true and unique person of value is important for human health and well-being (Eriksson & Lindström, 2000). Having to adapt to health care that is predetermined and inflexible can thus be experienced as unworthy by the older adult, who is a unique person. We argue that inflexible health care is not preferable, since health care and personnel should look instead for expressions of the unique inner world (c.f. Ueland et al., 2018) of the frail older adult, in order to take their longing into account in the process of enhancing and strengthening their well-being in daily life (cf. Pennbrant & Karlsson, 2019). Health-care professionals can confirm the older adults’ dignity by being present, and showing respect (Pennbrant & Karlsson, 2019). This can also be compared to person-centeredness, according to Edvardsson et al. (2014). Eriksson (1995) also states that health is viewed as a movement between the actual and potential capacities within a person, and on vitality of body, soul, and spirit, where their needs and desires, the will to find meaning, life and love constitute the source of energy that ultimately determines the direction of this movement.

## Methodological considerations

One limitation to this study might be that four different researchers conducted the interviews and these had all different experiences in conducting interviews. However, as earlier mentioned, the most experienced researcher (J.H.) with regards to qualitative studies and interview techniques conducted and transcribed most interviews and data. Also, the researchers had discussed and agreed upon how to conduct the interviews and transcribe the interviews. Another limitation is that no triangulation was undertaken since only one of the researchers performed the analysis, however, the researchers came together to discuss the results. A strength of this study is the number of participants (17) in the qualitative study. The sample in the study was considered appropriate and the fact that the participants were a heterogenous group of people, of both women and men, ensured the quality of the data collected and can be considered a strength in the study. The researchers enabled trust and a good relationship between themselves and the participants by letting them decide the terms and the phase of the interview. Voluntary participation and honesty as well as openness during the interview, created a good and trustful atmosphere (Whitney & Trosten-Bloom, 2019). The fact that the interviews were held in the frail older adults’ own homes demanded they be conducted in a dignified and respectful manner. This was managed, for example, by initially taking some time before the interview started to have a little small talk about the frail older adult’s home or interior, such as flowers, pets, or simply letting the participant talk about something that they themselves wanted to talk about with the interviewee. The researchers’ preunderstanding could have been considered a weakness in the sense that data could be overlooked. However, the preunderstanding was managed by constantly repeating it during the data analyses, which was seen as a strength of the study, since it was considered helpful in the understanding of the data on a deeper level. Another strength of this study was the fact that the interviews were individual interviews conducted in the frail older adults’ homes, which guaranteed a closeness and trusting relationship to develop. In this environment the participants felt safe to openly express themselves and to speak to the end, without being interrupted, and to have the opportunity to express whatever they wished. The fact that the participants discussed the themes in the findings several times contributed to the validity of the study.

## Conclusions and implications

This study contributes to the limited research available so far, concerning longing among frail older adults. More specifically, the study deepens the understanding of the underlying mechanisms of longing as an inner resource for setting the movement towards well-being into motion. This new understanding can help caregivers and social workers in understanding of what longings may trigger frail older adults’ movement towards well-being into motion. At the same time, we found that the older adults, due to frailty, are not always able to progress in the movement towards well-being and health, since frailty may be a limiting factor. Other persons, such as friends and staff, may be in a key position here to support this movement. This study also contributes with basic research in the development of the theory of Caring Science (Lindström et al., 2011). Further studies could focus on how other persons can support frail older adults to fulfil their longing in daily life.
